# Metastatic Pleomorphic Adenoma: A Systematic Review

**DOI:** 10.1007/s12105-025-01856-1

**Published:** 2025-11-20

**Authors:** Alberto Jose Peraza-Labrador, Doreen Palsgrove, Marcelo Villacis, Victoria Woo, Nestor R. Gonzalez, Andreas Ciscato, Justin Bishop

**Affiliations:** 1 Department of Diagnostic Sciences, Texas A&M University College of Dentistry. Dallas, 3302 Gaston Avenue, Texas, zip code 75246 USA; 2 Department of Pathology, University of Texas Southwestern Medical Center, 5323 Harry Hines Boulevard, Dallas, TX 75390 USA; 3 Oral Surgery Department, Dental School, Universidad Internacional de Ecuador, Quito, Ecuador; 4 Department of Otolaryngology, Military Hospital Bogota, Colombia, Bogotá, Colombia; 5 Department of Pathology, University of Texas Southwestern Medical Center, 6201 Harry Hines Blvd, Dallas, TX 75390 USA

**Keywords:** Metastasis, Pleomorphic adenoma, Benign mixed tumor, Salivary tumor

## Abstract

**Background:**

Metastatic pleomorphic adenoma (MPA) is a benign-appearing pleomorphic adenoma that spreads to regional and distant sites, such as lymph nodes, lungs, or bones, despite lacking malignant features histologically.

**Methods:**

A systematic review of documented case reports and case series of metastatic pleomorphic adenoma is presented. Searches were conducted across various databases to identify articles published from 1953 to December 2024. The variables included were gender, age, symptoms, type of gland, histological features, molecular studies, type of treatment, and follow-up.

**Results:**

Of the 95 cases reported, 58.8% affected females, and 41.1% were males. The average age for MPA was 49.9 years, and the mean size was 2.4 cm. The parotid gland was the most commonly affected, accounting for 75.8% of cases, followed by the submandibular gland with 13.6%. The treatment for the (benign) pleomorphic adenoma was excision with 42.1%, followed by superficial parotidectomy with 28.4%. The average time to MPA was 198 months. Among patients with follow-up, the bone and cervical lymph nodes were the most common sites of metastasis, with rates of 21.88% and 20.31% respectively. 52.7% of the patients were alive, and 8.4% died from the disease.

**Conclusion:**

MPA is a rare paradoxical entity in which a histologically benign salivary gland tumor exhibits malignant behavior by spreading to regional and distant sites, typically after several recurrences. Diagnosis requires a careful correlation of clinical history, histopathology, and radiographic imaging, including long-term follow-up after the surgery of Pleomorphic adenomas.

## Introduction

Pleomorphic adenomas (PAs) are the most common salivary gland neoplasm, accounting for approximately 60–80% of all salivary gland tumors [1, 2]. The parotid gland is the most frequently affected site, with approximately 85% of cases, followed by the minor salivary glands (10% of cases) and the submandibular glands (5% of cases) [3].

PAs of the parotid gland predominantly arise in the superficial lobe (about 80%), with 15–20% originating in the deep lobe and potentially extending into the parapharyngeal space, typically without symptoms or facial nerve involvement [4–6]. Standard management involves superficial or total parotidectomy with facial nerve preservation [7]. Recurrence rates are influenced by the surgical approach, with enucleation (88.9%) and simple excision (46.9%) carrying significantly higher risks compared to superficial (approximately 4%) and total parotidectomy (approximately 1%) [8, 9]. Recurrent PAs may present as multinodular lesions at or near the original site, with a 7% risk of further recurrence following reintervention [10]. Malignant transformation, though rare, occurs in 3% and may be signaled by rapid tumor growth or new-onset facial nerve dysfunction [11].

Foote and Frazell first described metastatic PA (MPA) in 1953 [12]. The WHO 5th edition classification recognizes Metastasizing Pleomorphic Adenoma (MPA), similar to the previous edition, as a rare salivary gland tumor, typically associated with multiple prior recurrences. Common metastatic sites include bone, lung, and cervical lymph nodes [13]. Despite its metastatic potential, no definitive histological or molecular markers currently predict such behavior [13, 14].

A highly speculative theory is that MPA may arise as a consequence of prior surgical interventions, potentially facilitating tumor cell entry into lymphatic or vascular channels and leading to regional or distant metastases, often years after the initial procedure [15, 16]. Hematogenous dissemination is the prevailing hypothesis, supported by the frequent occurrence of metastases in the lungs and bones. Alternative mechanisms, such as aspiration of shed tumor cells and lymphatic spread along the cervical nodal chain, have also been proposed, though less substantiated [17, 18].

MPA occurs between 1.5 and 55 years following initial surgical intervention of a PA [20]. The treatment of choice is surgical and has been shown to improve survival compared to nonoperative management [21]. According to WHO data, approximately 40% of patients succumb to the disease, with a five-year mortality rate approaching 50%. In contrast, 47% of patients achieve disease-free survival, while 13% continue to live with active disease [20].

This systematic review aims to provide a comprehensive update on the epidemiology, clinical, and molecular characteristics of MPA, with a focus on gender- and age-related differences in metastatic patterns and survival rates.

## Materials and Methods

A systematic review of the published literature on cases of MPA was performed. According to the guidelines, this study met the criteria for nonhuman subject research. As a result, institutional review board approval was not required.

### Search Strategy

**Fig. 1:**
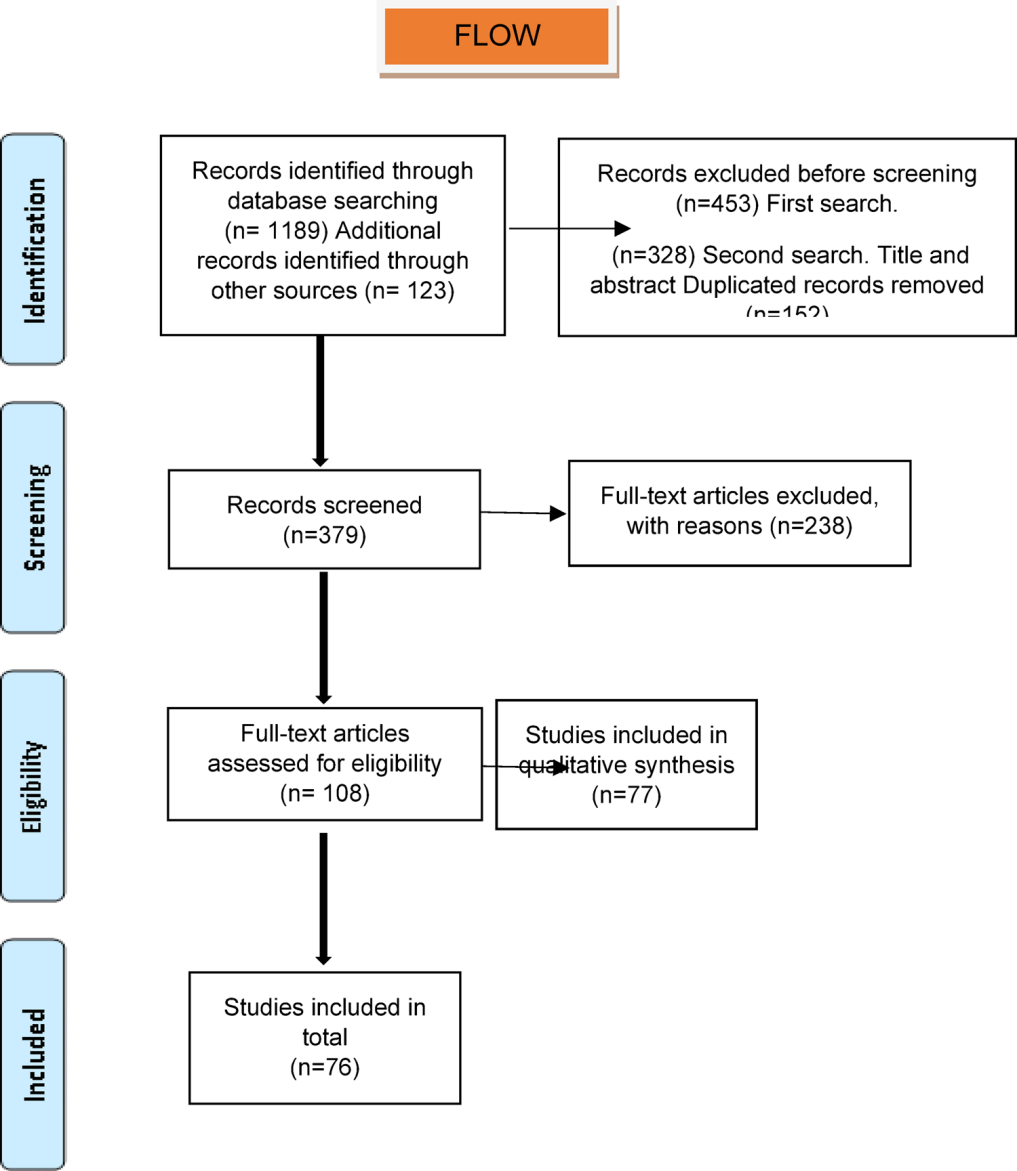
Flow chart of the selected papers

The research followed the PRISMA statement (Preferred Reporting Items for Systematic Reviews and Meta-analyses) [22]. An electronic search was conducted from 1953 until December 2024 (Fig. 1) from PUBMED, EMBASE, SCOPUS, and SCIENCE-DIRECT. The MeSH terms were “Metastatic pleomorphic adenoma” OR “Metastatic salivary benign mixed tumor” OR “Metastasizing pleomorphic adenoma” AND “Salivary gland metastasis.” A manual search was also made from referenced papers. A protocol was enrolled and recorded with the International Prospective Register of Systematic Reviews PROSPERO (CRD420251001423). The research question was based on the “PVO” framework for systematic reviews (P = patient/population, V = variable, O = outcome). This research aimed to answer the following question: What are the demographics, clinical features, and factors associated with metastasis of PA?

The certainty of the evidence from the primary studies will be assessed using pre-established strategies, such as evaluating methodological quality with the Oxford Centre for Evidence-based Medicine classification system and excluding studies deemed irrelevant by the reviewers. The findings will be presented in tables containing the most pertinent information.

### Selection Criteria and Risk of Bias

Studies with individual data for the diagnosis of primary pleomorphic adenoma and demographics of patients with MPA were included without age, gender, or anatomic location limitations. Exclusion criteria included non-English-language, animal, and cadaveric specimens, non-obtainable full-text studies, review studies, systematic reviews, and studies with insufficient or incomplete data. Two investigators (A.P.L. and A.C) independently reviewed the search to determine that all appropriate articles were included in the analysis. Any disagreements were resolved through discussion with a third author (JB). The type of studies included were case reports [14,17,18,26–30,34,35,39–42,44–48,50–59,63–68,70–72,80–84,86–95,97–99,102–109–113] and case series [12, 38, 43, 48, 69]. For conceptual purposes of this review, the types of surgery were defined as excision (as the surgical removal of only the tumor from the salivary gland, preserving as much of the surrounding parotid gland tissue as possible) [10]. Superficial parotidectomy (defined as the surgical removal of a portion of the gland, including the tumor that also involves the removal of some surrounding tissue to clear margins) [11], and total parotidectomy (which consisted of the complete surgical removal of the parotid gland, including both the superficial and deep lobes) [10, 11].

The strength of evidence for each of the included articles was assessed by the Oxford University Centre for Evidence-based Medicine (http:// www. cebm. net/ index. aspx?o = 1025). (Table 1) These guidelines rank the studies from level 1 (highest) to 5 (lowest): level 1 (randomized controlled trials), level 2 (prospective cohort clinical studies), level 3 (3A well-conducted case–control study with low risk of bias and a high probability of a causal relationship, and 3B a case–control or case report study with significant bias or confounding factors. Level 4 (case series studies) and level 5 (case-based reasoning and bench studies). To address missing data, this study employs the pairwise deletion method, analyzing only the available data points between each pair of variables [23]. For the selected cases, the published histologic data were reviewed by a Head and Neck Pathologist at the University of Texas Southwestern Medical Center in Dallas, Texas (AC), with evaluation of the primary pleomorphic adenoma, recurrences, and metastatic pleomorphic adenoma (MPA) when available. The qualitative analysis was supplemented with descriptive statistics derived from the raw data, maintaining the original measurement units. Data normality was evaluated using the Kolmogorov–Smirnov test. For variables following a normal distribution, results were presented as mean ± standard deviation; for non-normally distributed variables, the median and interquartile range (IQR) were reported. Precision was assessed using 95% Wald confidence intervals for normally distributed quantitative variables, and the Agresti–Coull method for proportions. Time-to-event outcomes, including recurrence and metastasis, were analyzed using Kaplan–Meier (KM) estimates for endpoints with consistently reported follow-up durations (i.e., time to first, second, and third recurrence and time to metastasis). All statistical analyses were conducted using Microsoft Excel 2018 (Microsoft Corp., Redmond, WA, USA) and the Statistical Package for the Social Sciences (SPSS) software, version 20.0 Copyright IBM (SPSS Inc., Chicago, IL, USA).

**Table 1:** Oxford classification. disease-related death (r)

Author/year	Study	Gender	Primary gland	Age for PA	Age for MPA	Organ with metastasis	Follow-up/months	Patient status	Evidence level
Foote F /1953	Case series	female	right Parotid	26	39	Lung	not mentioned	not mentioned	4
Fine G / 1961	Case report	male	right parotid	48	52	Vertebrae, humerus, ribs	12	died	3b
Clapp WA / 1966	Case report	male	right parotid	40	49	Thigh and muscle, shoulder, abdomen	7	died	3b
Youngs YR/1973	Case report	female	right Parotid	16	27	Liver	12	alive	4
Giltman L / 1977	Case report	male	right Parotid	23	58	scalp	1	alive	3b
Chen KT / 1978	Case report	female	right Parotid	39	51	sacrum	24	alive	3b
Ma, Wajed / 1978	Case report	female	right submandibular	38	56	vertebrae	not mentioned	not mentioned	4
Morrison PD / 1984	Case report	male	right Parotid	18	69	temporal bone	1	not mentioned	4
Drinkard DW / 1986	Case report	male	left parotid	16	33	mandible	12	alive	3b
Wermuth DJ / 1988	Case report	male	right soft palate	27	37	Lung	1	alive	3b
el-Naggar A / 1988	Case report	female	left parotid	54	63	Abdomen	12	not mentioned	3b
Collina G / 1989	Case report	female	right submandibular	26	35	Cervical lymph nodes	12	alive	3b
		male	right Parotid	8	14	Cervical lymph nodes	36	alive	
Cresson DH / 1990	Case report	female	right Parotid	77	84	Retroperitoneum	60	alive	3a
Sim DW / 1990	Case report	male	right Parotid	16	55	Lung	2	alive	3b
Freeman SB / 1990	Case report	female	left nasal septum	15	32	Cervical lymph nodes	12	alive	3b
Landolt U / 1990	Case report	male	right soft palate	73	75	Lung	not mentioned	not mentioned	4
Girson M / 1991	Case report	male	left parotid	17	33	Calvaria	not mentioned	not mentioned	4
Pitman MB / 1991	Case report	female	right Parotid	70	72	Lung, sacrum,	not mentioned	not mentioned	4
		male	right Parotid	23	63	Lung, vertebrae	not mentioned	not mentioned	
Wenig BM /1992	Case series	female	right Parotid	51	77	Kidney, skin	0	died	3b
		male	left submandibular	41	61	Sacrum, thorax, humerus	36	died (r)	
		female	left parotid	12	53	Skin, femur, central nervous system	24	died (r)	
		female	left parotid	72	83	Retroperitoneum	60	alive	
		female	right Parotid	24	33	Lung	60	alive	
		male	right parotid	15	30	Cervical lymph nodes	0	alive	
		female	left nasal septum	13	31	Cervical lymph nodes	36	alive	
		male	left submandibular	14	20	Cervical lymph nodes	192	alive	
		male	left parotid	52	76	Lung	0	died	
		male	left parotid	17	33	Calvaria	24	alive	
		female	right Parotid	34	65	Kidney	0	alive	
Qureshi AA / 1994	Case report	male	left parotid	16	34	Supraspinatus muscle, shoulder	12	alive	3b
Olsha O / 1995	Case report	male	left parotid	26	46	Liver	48	alive	3b
Schreibstein JM / 1995	Case report	female	right parotid	50	75	Sacrum	1	alive	4
Klijanienko J / 1997	Case report	female	hard palate	56	63	Lung, cervical lymph nodes	16	died (r)	3b
		male	parotid	53	55	vertebrae, skull base	12	died (r)	
Hoorweg JJ / 1998	Case series	female	left parotid	45	73	Lung	36	alive	3a
		male	left parotid	30	41	Scalp	108	alive	
		female	right submandibular	35	46	Sacrum, mandible	18	alive	
Sampson BA / 1998	Case report	male	right parotid	72	74	Lung, sacrum, sphenoid	6	died (r)	3b
Goodisson DW / 1999	Case report	female	left hard palate	13	20	Maxillary bone	24	alive	3b
Czader M / 2000	Case report	female	right parotid	53	56	Lung, kidney, skull base	6	alive	3b
Chen IH / 2000	Case report	female	left parotid	22	51	Cervical lymph nodes	10	alive	3b
Hay MA / 2001	Case report	female	left parotid	36	51	Cervical lymph nodes,	26	alive	3b
Raja V / 2002	Case report	male	left parotid	67	72	Lung	not mentioned	not mentioned	4
Marioni G / 2003	Case report	male	right parotid	20	32	Sinonasal	6	alive	3b
Khademi / 2003	Case report	female	tongue left area	19	19	Cervical lymph nodes	24	alive	3b
Yoshizaki T / 2004	Case report	female	left parotid	10	33	Lung	21	alive	3b
Muthusami J.C. / 2006	Case report	male	right Parotid	23	31	Scalp	24	alive	3b
Steele NP / 2007	Case report	female	right Parotid	15	43	Lung	1	not mentioned	4
Sabesan T/ 2007	Case report	male	left parotid	33	60	Supraclavicular fossa	24	alive	3b
Hyun Yee H / 2007	Case report	female	left submandibular	28	68	Lung, skin, brain	11	not mentioned	4
Van der Schroeff MP / 2007	Case report	female	right parotid	19	62	Ribs	6	died	3b
Sit KY / 2008	Case report	male	left submandibular	38	46	Lung	6	alive	3b
Rodriguez FJ / 2008	Case report	female	left parotid	54	57	Lung	60	died (r)	3b
Ghosh A / 2008	Case report	female	left parotid	29	35	Shoulder,	not mentioned	not mentioned	4
Xiao L / 2008	Case report	female	left parotid	46	72	Sacrum, kidney	34	died (r)	3b
Ebbing J / 2009	Case report	female	left parotid	20	49	Kidney	not mentioned	not mentioned	4
Zhang Y / 2009	Case report	female	left parotid	45	64	Lung	1	alive	3b
Larbcharoensub / 2009	Case series	female	right submandibular	36	45	Cervical lymph nodes	6	alive	3a
		female	left parotid	32	42	Cervical lymph nodes	30	alive	
		female	right parotid	27	40	Cervical lymph nodes	1	alive	
Bhutta MF / 2010	Case report	female	left parotid	62	75	Kidney	9	alive	3b
Bae CH / 2010	Case report	male	soft palate	48	49	Sphenoid sinus	not mentioned	not mentioned	4
Singhal A / 2010	case report	male	left parotid	16	46	liver	not mentioned	not mentioned	4
Chen CP / 2011	Case report	male	right buccal mucosa	73	75	Lung, cervical lymph nodes	44	not mentioned	4
Reiland MD / 2012	Case report	male	left parotid	15	31	Scalp, maxillary bone, infratemporal fossa, sinus	132	alive	3a
Vivian MA / 2012	Case report	female	right Parotid	24	40	Kidney	not mentioned	not mentioned	4
Santaliz R /2012	Case report	female	left submandibular	23	53	Cervical lymph nodes	10	alive	3b
RF Chinoy / 2013	case report	male	parotid	50	50	Cervical lymph nodes	not mentioned	not mentioned	4
Akiba J / 2013	case report	female	left hard palate	42	62	Maxillary bone	not mentioned	not mentioned	4
Miladi S / 2014	Case report	female	left submandibular	18	38	Cervical lymph nodes	8	alive	3b
Tarsitano A / 2014	Case report	female	left parotid	9	11	Sinonasal	6	alive	3b
Abou-Foul AK / 2014	Case report	male	left parotid	50	57	Lung, liver	16	alive	3b
McGarry JG / 2015	Case report	female	right Parotid	40	65	Shoulder,	3	died (r)	3b
M Moonim / 2015	Case report	female	right Parotid	16	68	Liver, thyroid	6	alive	3b
Mariano FV / 2015	Case report	female	left parotid	65	68	Scalp	not mentioned	not mentioned	4
Young VS / 2015	Case report	female	parotid	45	65	Liver	3	not mentioned	4
Knight J / 2017	Case report	male	right submandibular	62	68	Vertebrae	7	not mentioned	4
Nakai A /2017	Case report	female	right Parotid	28	38	Lung	3	not mentioned	4
Koyama M/ 2018	Case report	female	left parotid	40	61	Lung, kidney, skull base	60	alive	3b
Koyama M / 2018	Case report	female	left parotid	40	63	Lung, liver, kidney, sinonasal, brain	60	alive	3b
Wasserman JK / 2019	Case series	female	parotid	35	57	Thigh and muscle	not mentioned	not mentioned	4
		male	parotid	36	57	Cervical lymph nodes	not mentioned	not mentioned	
		male	parotid	43	48	vertebrae	not mentioned	not mentioned	
		male	parotid	34	59	Cervical lymph nodes	not mentioned	not mentioned	
Wong DKC / 2019	case report	female	right Parotid	31	61	Cervical lymph nodes, infratemporal fossa	6	alive	3b
Shoukair FL / 2020	case report	female	right submandibular	13	37	cervical lymph nodes, lips	24	alive	3b
Fonseca D / 2022	Case report	female	right parotid	14	29	Cervical lymph nodes	1	not mentioned	4
Lu H / 2023	Case report	male	left submandibular	28	34	Cervical lymph node	6	alive	3b
Lobo J / 2023	Case report	female	parotid	12	43	Kidney	24	alive	4
Catarsi L /2023	Case report	female	left parotid	23	31	Cervical lymph nodes	not mentioned	not mentioned	4
AlNahwe RW / 2023	Case report	male	right Parotid	25	39	Scalp	24	not mentioned	4
Tsai /2024	case report	female	left parotid	61	61	Cervical lymph nodes	126	not mentioned	4
Jacob S / 2024	case report	male	left parotid	12	19	cervical lymph node	not mentioned	not mentioned	4

## Results

Some of the highlights are presented here. The complete results and data of the 95 cases are summarized in Tables 2, 3 (Demographics), and Tables 4, 5, 6, 7 (Kaplan–Meier).

**Table 2:** Demographics of metastatic pleomorphic adenoma. *[IC 95%] two-sided confidence intervals calculated using the Agresti–Coull method; total n = 95 cases. Mean ± standard deviation is reported for normally distributed variables (Kolmogorov–Smirnov test *p* > *0.05*)

Total	100% (N = 95) Total cases	
Age (years) Age at PA Diagnosis Age at the time of Metastasis	Mean ± SD (Min/Max)	[CI95%]
	33.7 ± 17.9 (8/77) 49.9 ± 17.1 (11/84)	[30.1–37.3] [46.5–53.3]
Size of MPA lesion (cm)	11.8 ± 17.9 (1/145)	[8.2–15.4]
	100% (n = 5)	[CI95%]
Sex		
Female	58.8% (n = 56)	[50.0–69.6]
Male	41.1% (n = 39)	[30.4–50.0]
Type of gland primary		
Parotid	75.8% (n = 72)	[66.4–83.2] *
Submandibular	13.6% (n = 13)	[8.2–22.2] *
Bucogingival	1.0% (n = 1)	[0.2–5.6] *
Soft palate	3.2% (n = 3)	[1.1–8.7] *
Nasal septum	2.2% (n = 2)	[0.6–5.6] *
Hard palate	3.2% (n = 3)	[1.1–8.7] *
Tongue	1.0% (n = 1)	[0.2–5.6] *
Anatomic location		
Right	43.2% (n = 41)	[33.4–53.2]
Left	46.4% (n = 44)	[36.5–56.3]
Not mentioned	10.6% (n = 10)	–
Type of surgery		
Excision	42.1% (n = 40)	[33.4–53.2]
Superficial parotidectomy	28.4% (n = 27)	[19.6–29.2]
Total parotidectomy (resection)	17.9% (n = 17)	[13.2–23.1]
Enucleation	8.4% (n = 8)	[2.8–13.7]
Fragmented excision	1.0% (n = 1)	[0.0–6.2] *
Not mentioned	2.1% (n = 2)	–
	Mean ± SD (n)**	[CI95%]
Recurrence—First (months)	87.1 ± 92.1 (n = 62)	[64.4–109.8]
Recurrence—second (months)	100.3 ± 69.5(n = 29)	[75.0–125.6]
Recurrence—third (months)	149.4 ± 92.3(n = 12)	[97.2–201.6]
Recurrence—fourth (months)	105.6 ± 31.04(n = 6)	[80.8–130.4]
Recurrence—fifth (months)	142.5 ± 12.3(n = 4)	[130.4–154.5]
Duration time for metastasis (months)	198.3 ± 138.3(n = 95)	[170.5–226.1]
Follow-up (months)	26.9 ± 33.7(n = 69)	[19.0–34.9]
	100% (n = 95)	[CI95%]
Patient status		
Alive	52.7% `(n = 50)	[41.6–61.5]
Dead from the disease	8.4% (n = 8)	[6.6–20.2]
Dead from another condition	5.3% (n = 5)	[1.9–12.03]
Not mentioned	34% (n = 32)	–

**Table 3:** Organs affected by metastatic pleomorphic adenoma. *[IC 95%] two-sided confidence intervals calculated using the Agresti–Coull method; total (n = 128) lesions from 95 cases

	128 = 100% (N = 95)	[CI95%]
Bone (All sites)	21.88% (n = 28)	[15.5–29.8]
Sacrum/ iliac bone	5.47% (n = 7)	[2.5–11.1]
Vertebrae	4.69% (n = 6)	[2.0–10.1]
Maxillary bone (and palate)	2.34% (n = 3)	[0.5–7.0]
Infratemporal fossa or temporal bone	2.34% (n = 3)	[0.5–7.0]
Skull base	1.56% (n = 2)	[0.1–5.9]
Mandible	1.56% (n = 2)	[0.1–5.9]
Humerus	1.56% (n = 2)	[0.1–5.9]
Ribs	1.56% (n = 2)	[0.1–5.9]
Femur	0.78% (n = 1)	[0.0–4.7]
Cervical lymph nodes	20.31% (n = 26)	[14.2–28.2]
Lung	17.97% (n = 23)	[12.2–25.6]
Kidney	7.81% (n = 10)	[4.1–13.9]
Liver	5.47% (n = 7)	[2.5–11.1]
Scalp	4.69% (n = 6)	[2.0–10.1]
Sinonasal	3.12% (n = 4)	[1.0–8.0]
Supraspinatus muscle, scapula, or shoulder	3.12% (n = 4)	[1.0–8.0]
Skin	2.34% (n = 3)	[0.5–7.0]
Brain	1.56% (n = 2)	[0.1–5.9]
Sphenoid sinus	1.56% (n = 2)	[0.1–5.9]
Upper and lower lip	0.78% (n = 1)	[0.0–4.7]
Thyroid	0.78% (n = 1)	[0.0–4.7]
Supraclavicular fossa	0.78% (n = 1)	[0.0–4.7]
Abdomen	1.56% (n = 2)	[0.1–5.9]
Retroperitoneum	1.56% (n = 2)	[0.1–5.9]
Thigh and muscle	1.56% (n = 2)	[0.1–5.9]
Central nervous system	0.78% (n = 1)	[0.0–4.7]

**Table 4:**
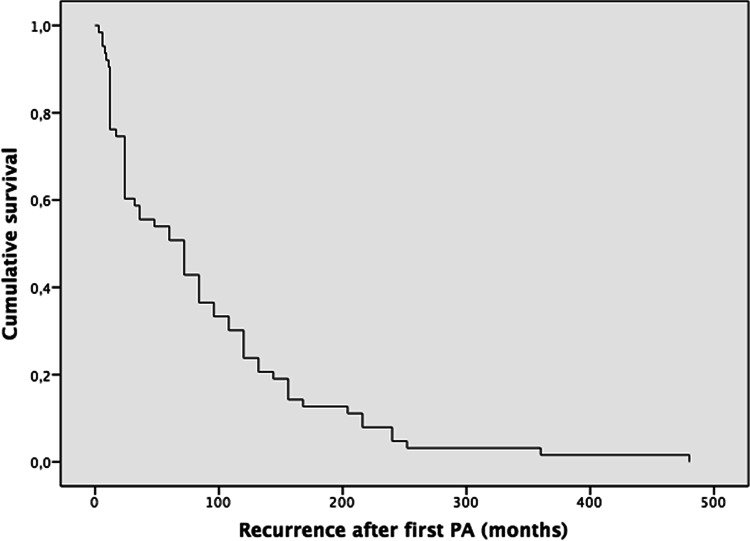
A Kaplan–Meier (KM) curve was constructed to assess time to first recurrence following initial PA, with follow-up extending to nearly 500 months

*Recurrence-First (months) Median recurrence 72 Mean 87.1 ± SD 92.1 (n=62) IC95% [64.4–109.8]

**Table 5:**
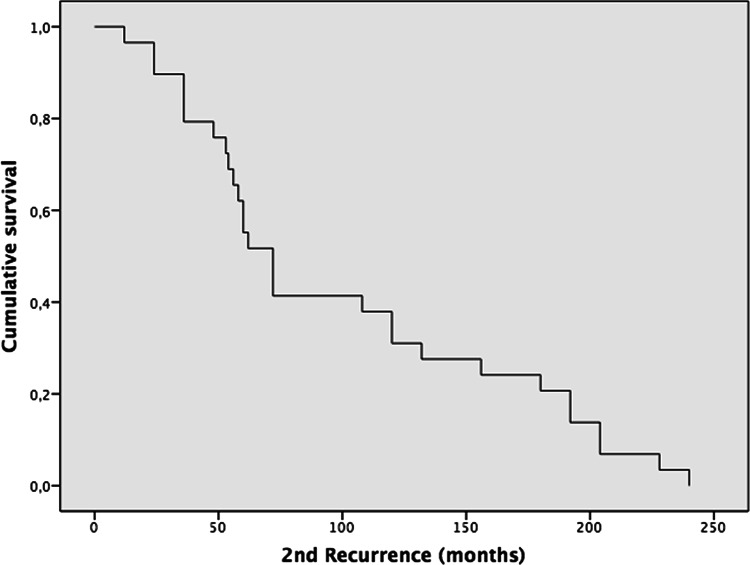
The second recurrence–free survival function was estimated with KM, with a maximum follow-up period of approximately 240 to 250 months

*Recurrence–2nd (months) Median recurrence 72 Mean 100.3±69.5 (n=29) IC95% [75.0–125.6]

**Table 6:**
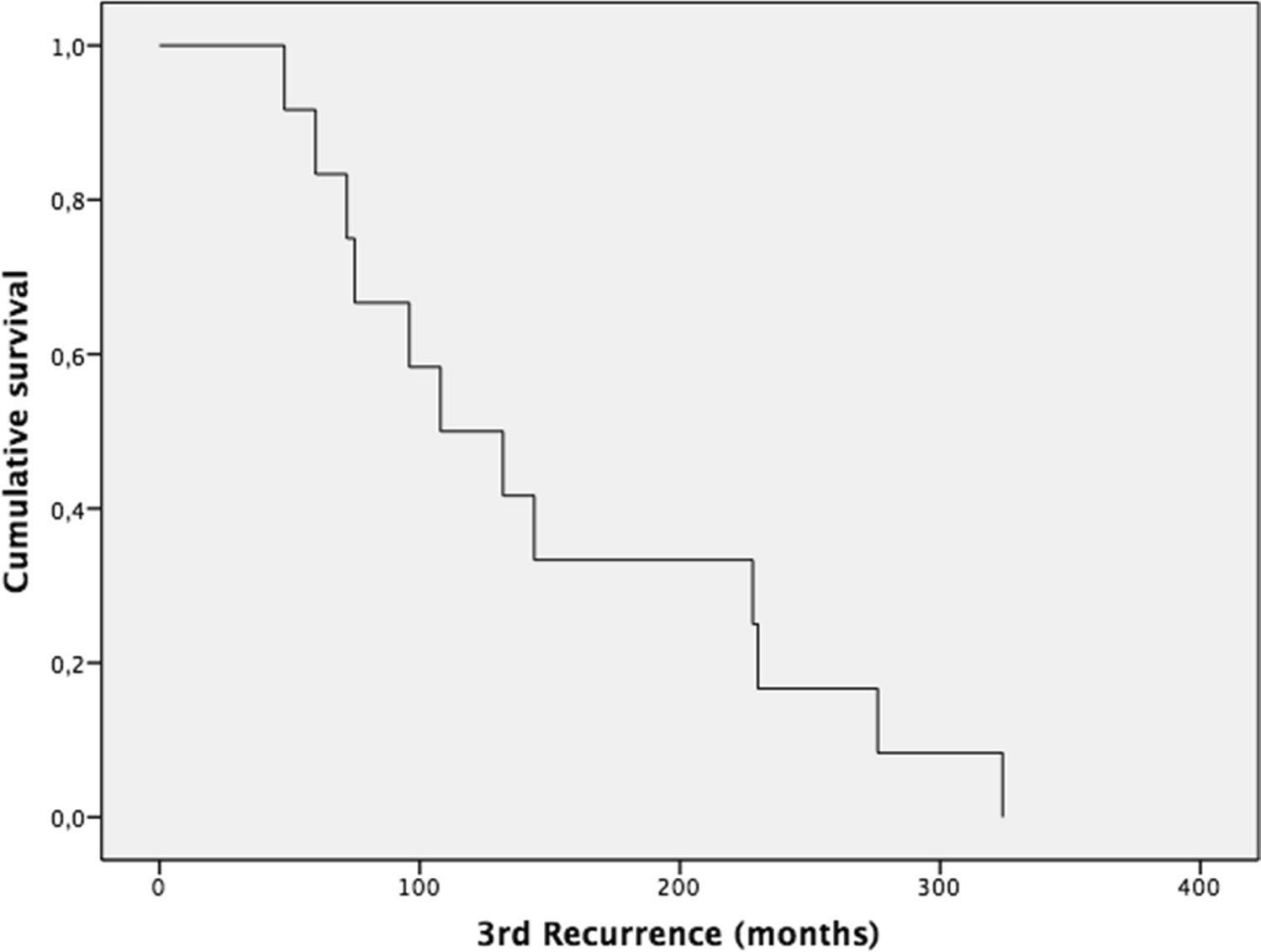
Third recurrence-free survival. The KM curve demonstrates a stepwise decline without a plateau, with a concentration of events occurring between 50 and 140 months

*Recurrence–3rd (months) Median recurrence 108 Mean 149.4±92.3 (n=12) IC95% [97.2–201.6]

**Table 7:**
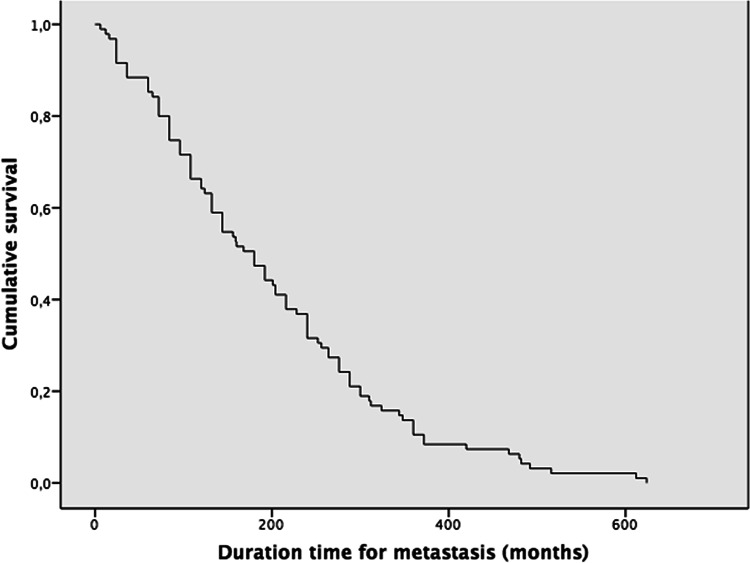
KM curve for time to metastasis reveals a sustained decline in metastasis-free survival (MFS), without reaching a plateau throughout the follow-up period

*Metastasis–time to event (months) Median recurrence 180 Mean 198.3 ± 138.3 (n=95) IC95% [170.5–226.1]

Age of PA diagnosis: The patients’ ages ranged from 9 to 77 years. The average age of diagnosis for both genders was 33.7 ± 17.9. For females, the average age was 32.98 years, with the youngest female patient diagnosed at 9 years [34]. For males, the average was 33.79 years, with the youngest diagnosed at 8 years [35].

Type of surgery and recurrence: The most common treatment for PAs was excision in 42.1% (n = 40) cases. The next most common treatment was superficial parotidectomy with 28.4% (n = 28) cases. Seventeen (n = 17), 17.9% of patients received a total parotidectomy/resection. Recurrences: Several patients experienced recurrence of their PA (up to five recurrences) before developing metastasis. The mean time to the first recurrence was 87.1 ± 92.1 months (n = 62), while the second recurrence occurred after an average of 100.3 ± 69.5 months (n = 29). Subsequent recurrences were observed in only a small number of cases.

Age of MPA diagnosis: The average age of diagnosis for both genders was 49.9 ± 17.1; the youngest female patient was diagnosed at 11 years [34]. The youngest male patient was 20 years old [43].

Time to metastasis: The average time for MPA was 198.3 ± 138.3 months (n = 93), ranging from 0 to 624 months. In two cases, the MPA was diagnosed synchronously as the pleomorphic adenoma.

Patient status: From the follow-up data analyzed, 52.7% (n = 50) were alive with the disease, 8.4%(n = 8) had died from the MPA, and 5.3% (n = 5) had died for other reasons. Follow-up information was not available in 34% (n = 32) of cases.

Organ/site involved in MPA: To ensure a more accurate representation of metastatic distribution, the analysis included all affected organs rather than calculating metastasis rates solely on a per-patient basis. This method accounted for cases with multiple simultaneous metastatic sites and helped prevent inflation of metastasis percentages. Among the 95 cases reviewed, a total of 128 metastatic sites were documented. The most frequently affected organs were the bone and lymph nodes, at 21.88% (n = 28) cases and 20.31% (n = 26) of cases, respectively. Within the bone category, the sacrum (5.47%) and vertebrae (4.69%) were the most commonly involved sites, followed by the lungs, in 17.97% (n = 23) of cases. Interestingly, the kidney was involved in 7.81% (n = 10) of cases. The complete results and data of the organ/site affected by metastasizing pleomorphic adenoma of the salivary gland are summarized in Table 3.

## Discussion

Our systematic review included 76 studies, encompassing a total of 95 cases with a diagnosis of metastatic pleomorphic adenoma (MPA). Malignant transformation is a recognized negative outcome of pleomorphic adenomas (PAs), occurring in approximately 1.5% of cases within the first five years and rising to 9.5% after 15 years or more [24]. Among the resulting malignancies, salivary duct carcinoma is the most frequently reported subtype, accounting for 41% of cases [25]. In contrast, metastatic pleomorphic adenoma (MPA) represents an even rarer phenomenon of malignant transformation, with a capacity for regional or distant spread. Notably, MPA retains the histological features of a benign tumor, lacking cytological signs of malignancy such as cellular atypia, elevated mitotic activity, or necrosis. Diagnosis of MPA is therefore based solely on the presence of metastasis, despite the absence of malignant histopathological characteristics [13, 20].

### Tumor Recurrence

In our review, local recurrence for PAs occurred in 65% of cases, and multiple recurrences (from 1 to 5 recurrences) in 35% before the development of the MPA. The average time to the first recurrence was 87.1 months, and the average time to the second recurrence was 100.3 months (Table 2). Something unexpected was the recurrence after total parotidectomy, with 12.3%, where the average is 3% for primary PA [114]. The local and multiple recurrence rates in our study were similar to the review by Knight et al., who reported local recurrence in 72.8% and multiple recurrence in 37% of their cases [19]. The literature also documents a latency period between primary PA to MPA of 6 months [26] to over 52 years [27]. Our review showed that the mean age for PA was 33.7 years, with a range from 8 [35] to 77 [39]. For MPA, the mean age was 49.9 years, with a range from 11 [34] to 84 years [39]. Of note, there were 2 cases where the metastatic PA was diagnosed at the same time as the primary PA that had no previous surgical intervention [28, 29] (Table 4, 5, 6).

### Molecular Data

Although limited, several studies have reported significant findings related to the potential molecular mechanisms involved in metastasis. Notably, a study by Mariano et al. identified deletions in chromosomes 3p in PA and 9p in cases of MPA [30]. Chromosome 3p deletions have been implicated in various malignant epithelial neoplasms, including clear cell renal cell carcinoma, where deletions of 3p and 9p were observed in 25% and 47% of metastatic cases, respectively [31]. Furthermore, deletions in 9p have demonstrated a statistically significant association with both lymph node involvement and distant metastasis (*P* = *0.03*) [32].

Yu et al. identified the CD105 molecule in recurrent and metastasizing pleomorphic adenomas [36]. This molecule, also known as endoglin, is a transmembrane glycoprotein primarily expressed on endothelial cells, and it plays a significant role in metastasis. Elevated levels of CD105 have been detected in various cancers and are positively correlated with tumor progression and metastasis [37]. Two studies reported PLAG1 mutations in cases of MPA [38, 95]. In a study by Agaimy et al., six tumors were classified as conventional PAs, while four exhibited canalicular-trabecular morphology. Among these, three MPAs demonstrated HMGA2 gene fusions, four showed PLAG1 alterations, and one case harbored an NFIB mutation [96]. Akiba et al. found an MPA with PLAG1 expression with no gene rearrangements by (RT)-PCR. RT-PCR did not demonstrate any fusion genes, including PLAG1-CTNNB1, PLAG1-CHCHD7, PLAG1-LHIT, and PLAG1-TCEA1, although the internal control, Porphobilinogen Deaminase (PBGD), was identified in both primary and metastatic lesions [90].

Wasserman et al. identified a novel *HMGA2::-TMTC2* fusion in an MPA, representing a unique genetic alteration. This fusion was confirmed through RNA sequencing and fluorescence in situ hybridization (FISH) [38]. TMTC2 (Transmembrane O-Mannosyltransferase Targeting Cadherins 2) is located on chromosome 12q21.31. It is a protein that modifies cadherins, which are cell adhesion molecules [33]. These data support growing evidence that specific genetic alterations may contribute to the metastatic potential of PAs. The molecular events that underpin MPA merit additional investigation, as a greater understanding may have impacts on diagnosis and treatment.

### Mechanism of Spread

**Fig. 2:**
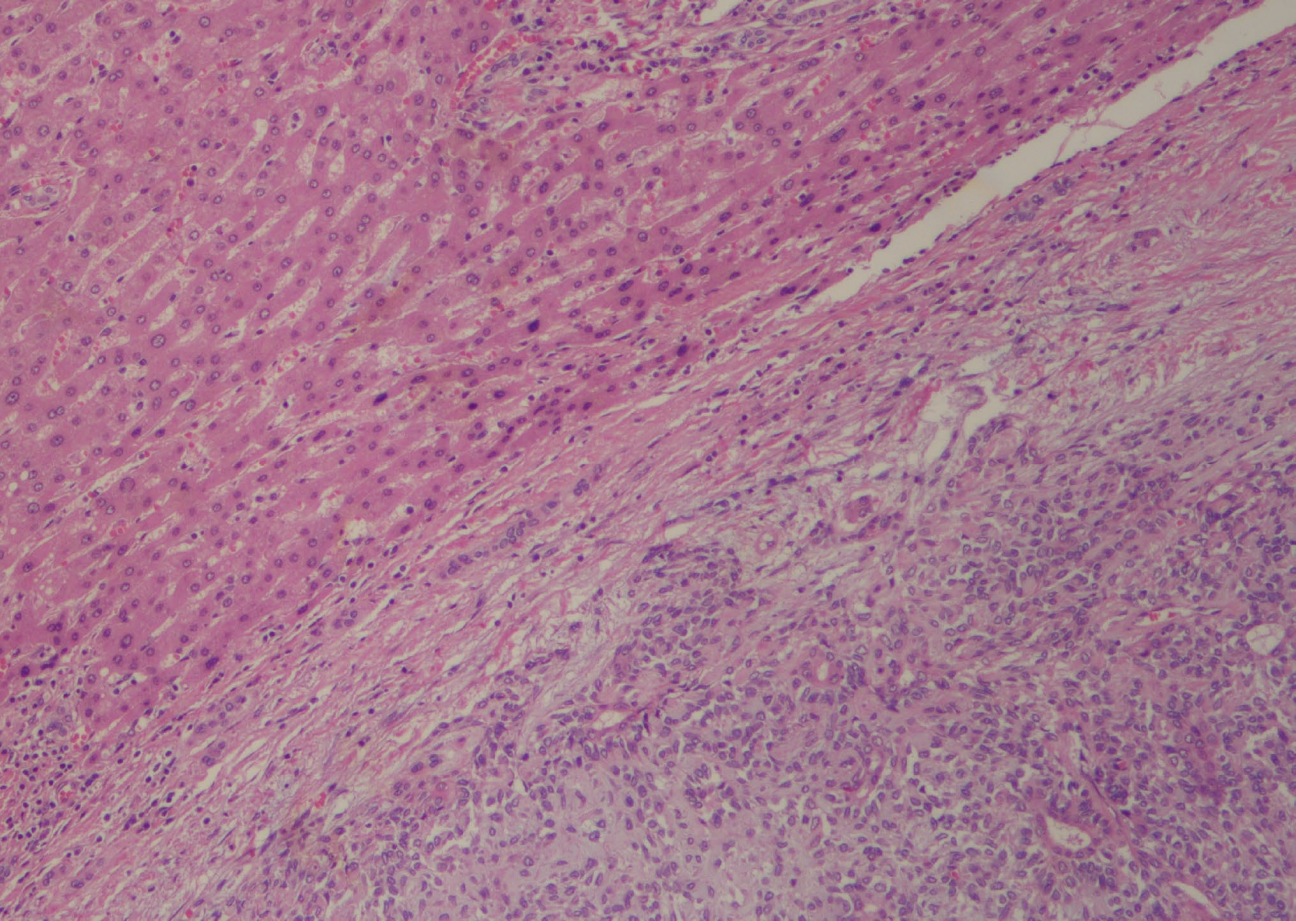
H&E 10x. Metastatic pleomorphic adenoma to the liver (courtesy of Columbia University Department of Pathology). Clear separation of hepatocytes and myoepithelial cells with chondroid and myxoid background

The route by which pleomorphic adenoma metastasizes remains poorly understood. A strong consideration (still hypothetical and speculative) has been given to hematogenous dissemination during the incisional biopsy or surgical manipulation (seeding) [16, 17]. Including fine needle aspiration [60]. The tumor may then form tumor embolisms in capsular blood vessels before hematogenous spread to different anatomical sites [61] (Fig. 2). Knight et al. reported that the most frequently affected sites in MPA were bone (36.6%), followed by the lungs (33.8%) and cervical lymph nodes. However, taking bone as an example, we found that most MPAs affect the axial skeleton, including the skull, vertebrae, ribs, sternum, sacrum, and mandible. MPA can be localized, affecting a specific bone [19, 56, 81, 86, 89–91, 93, 94], or multifocal, affecting multiple discrete bones [43, 47, 57, 80–84, 92]. In our study, MPA metastasized to bone in 21.88% of cases, being the sacrum 5.47% [38, 43, 57, 86–88] and the vertebrae in 4.69% [19, 38, 47, 56, 82, 89], the most common, followed by 11.72% in other bones. In the context of bone metastasis, pleomorphic adenoma-derived myoepithelial cells have been shown to secrete key bone-modulating factors, including Receptor Activator of Nuclear Factor Kappa-Β Ligand (RANKL) [76], Parathyroid Hormone-related Protein (PTHrP) [78], and Matrix Metalloproteinases (MMPs), particularly MMP-2 and MMP-9 [77]. RANKL and PTHrP play critical roles in bone metabolism and are frequently associated with underlying tumor-induced bone metastases. In pleomorphic adenomas, PTHrP expression has been observed in the inner lining of tubuloductal and cyst-like structures, single-layered ducts, isolated tumor cells, and clusters demonstrating squamous metaplasia [79].

For lymphatic spread, the second most common after bone, our study found 20.31% of cases [17, 26, 28, 29, 35, 38, 41, 43, 47, 52, 63–72]. A possible theory is that tumor cells may enter the lymphatic system during surgical procedures and subsequently reach distant sites such as the lungs. However, careful diagnostic evaluation is essential to avoid misclassifying a recurrent pleomorphic adenoma (PA) as an MPA, as some PAs may be mistakenly interpreted as lymph node metastases. True lymphatic metastasis is typically supported by cases occurring several years after the initial or repeated surgical interventions. Drainage to the deep cervical lymph nodes eventually enters the thoracic duct (main lymphatic channel). The thoracic duct empties into the left subclavian vein. Cells then follow the same systemic circulation [74].

**Fig. 3:**
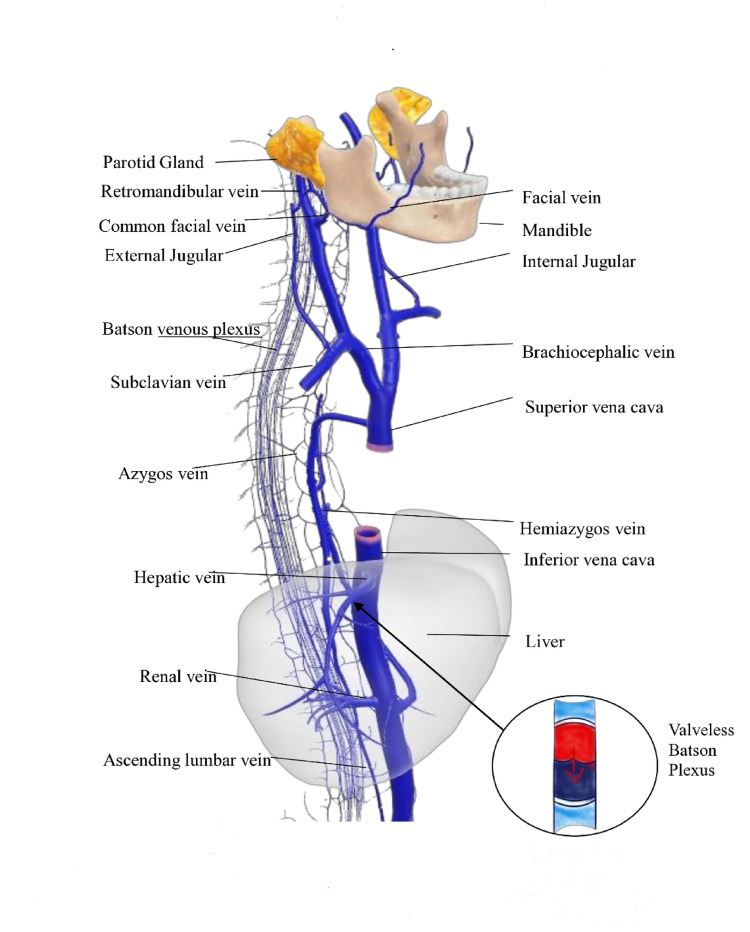
Routes of possible metastasis of MPA. The MPA could invade veins from the parotid region, which drain into the external and internal jugular veins, to the subclavian vein and the superior vena cava (SVC). Blood subsequently travels to the right side of the heart, specifically the right atrium and ventricle, where the tumor cells may survive in the lung’s capillary bed and enter the left side of the heart. through the lungs [74]. From there, it’s pumped into the aorta and distributed throughout the body via arteries. and out to the rest of the body. The Batson plexus plays a fundamental role. For example, the liver has a dual supply, with 25% of the blood from the hepatic artery (a branch of the aorta via the celiac trunk). Thus, the tumor cells can reach the liver through the hepatic artery and lodge in the liver’s capillary beds (sinusoids) [73]

The lungs were the third most common organ affected by MPA, in 17.97% of cases. This is interesting as the lung represents the first capillary bed encountered by venous blood draining from the head and neck, particularly via the facial and jugular veins. They also serve as a primary site for filtration and entrapment of circulating tumor cells within the pulmonary vasculature [62]. A key process for different metastasis is the Batson’s Venous Plexus (Vertebral Venous Plexus), which is a valveless venous network connecting pelvic, thoracic, and cranial veins to the vertebral column, including the sacrum. As the plexus is valveless, blood flow (and tumor cells) can reverse in direction during straining, coughing, or increased intra-abdominal pressure [75] (Fig. 3). On the other hand, Wermuth et al. have suggested that the oral cavity may serve as a potential source of pulmonary metastasis through the aspiration of tumor cells [40]. Even though 23 lung metastases were documented [12, 40–46, 48–59], four of 5 intraorally PA cases metastasized to the lung [40, 41, 47, 55], which may support this hypothesis.

While it is not possible at this time to predict which PA will metastasize, factors that may play a role in metastasis include multiple recurrences of PA, incomplete surgical excision (e.g., enucleation versus excision or parotidectomy), Long-standing tumors, and a history of capsular rupture during surgery.

### Prognosis

Although the overall mortality was 13.4% (n = cases), MPA-related deaths were 8 cases; some of the deaths occurred at 3 months [80] and 8 years after diagnosis of MPA [47]. Among those with comprehensive information, MPAs presenting with multiple metastases involving bone were associated with an 85% increased risk of death, with an average survival time of 13.3 months [43, 47, 57, 80–84]. This survival duration is comparable to the average of 9.8 months observed in head and neck metastases originating from other primary sites [85] (Table 7).

Of note, adjuvant radiotherapy was employed in several recurrent PAs and MPAs in our review [12, 18, 38, 44, 46–48, 50, 52, 53, 56–58, 63, 65, 69, 80, 81, 86, 98, 99, 101, 105, 108, 111]. However, it is challenging to determine whether radiation therapy improved survival or prognosis, as noted in a previous study [19].

Pleomorphic adenomas exhibit unpredictable latency and potential for regional or distant spread, necessitating long-term patient monitoring—especially in cases of incomplete excision or recurrence. Complete surgical removal with clear margins remains critical to reducing metastatic risk. Emerging perspectives suggest that PAs may possess low-grade malignant potential in specific contexts. To better understand MPA, future research should focus on molecular characterization through genomic and transcriptomic analyses. Establishing centralized registries or multi-institutional databases for rare salivary gland tumors could enhance data collection, supporting improved diagnostic and therapeutic strategies.

### Study Limitations

The primary limitation of this review lies in the variability of the available literature, as the majority of included cases were isolated reports or small case series, many of which lack standardized diagnostic criteria or uniform follow-up (Table 8). Furthermore, the infrequency of MPA likely contributes to underrecognition and diagnostic misclassification, potentially skewing incidence and clinical outcomes estimates. Nevertheless, by compiling findings across diverse sources, this review offers a broader perspective on the clinical presentation and pathological features of MPAs.

**Table 8:** Evaluation of the histologic features of different analyzed studies to evaluate the supporting images of the diagnosis and the included information for confirmation

Author	Year	PA Diagnosis	Diagnosis of recurrence	Diagnosis of MPA with PA histology	Comments
Foote, F. W	1953	correct	Histology of recurrence	PA not showing the background organ	black and white image
Marshall G	1961	Histology of primary parotid	Histology of local recurrence	Most likely recurrence	Black and white picture
Chen KT	1978	Primary compatible with PA	No histology	PA without the background of the organ	
El naggar	1988	Histology of primary parotid	Histology of local recurrence	PA not showing the background organ	Black and white picture
Wermuth DJ	1988	Histology of primary	No histology	PA not showing the background organ	
Guido Collina	1989	Histology of submandibular PA	No histology	Most likely met within the lymph node	Black and white
David Sim	1990	Histology of primary parotid	Histology of recurrence	PA in the lung	Black and white picture
Cresson	1990	Histology of primary parotid	No histology	MPA does not show the background organ	Black and white picture
Wenig BM	1992	Primary Hx, concerning for malignancy	No histology	Concerning for an EX PA-carcinoma	Black and white pictures, 5 cases shown
Jerzy Klijanienko	1997	Histology of primary parotid	Histology of recurrence	PA without background of the met- questionable met VS recurrence	pictures not optimal
JJ Hoorweg	1997	Histology of primary parotid	Histology of local recurrence	The picture is difficult to see	black and white picture
Goodison	1999	maxillary PA	Histology of recurrence	Most compatible with recurrence- not met	Black and white picture
Chen Ih	2000	Parotid		No good picture, difficult to evaluate	One black and white picture- not convincing
Marioni G	2003	Histology of primary parotid	Multiple cases included	PA not showing the background organ	CD105 positive
Yoshizaki T	2004	Histology of primary parotid	Histology of local recurrence	PA not showing the background organ	Black and white picture
Muthusami J.C	2006	Histology of primary parotid	Histology of local recurrence	Subcutaneous scalp PA	
Steele NP	2007	Histology of primary parotid	Histology of local recurrence	MPA is not showing the background organ	
T Sabesan	2007	Histology of primary parotid	No histology of recurrence	MPA in a supraclavicular lymph node	
Van der Schroeff	2007	Histology of primary parotid	Histology of local recurrence	MPA not showing the background organ	Black and white picture
Hyun Yee H	2007	Histology of submandibular PA	Histology of chondromyxoid syringoma	MPa in bone	
Xiao L	2008	No information		Hypercellular area of the PA concerning	no primary
Sit KY	2008	Histology of PA	No image	no image	
Rodríguez-Fernández J	2008	Histology of primary parotid		PA without the background of the met	black and white picture
Ebbing J	2009	Histology of primary parotid	No histology	MPA in the kidney	
Zhang Y	2009	Histology of primary parotid	Histology of recurrent multiple	Only cytology pictures—no background	only cytology pictures
Bhutta MF	2010	Histology of primary parotid	Histology of local recurrence	Picture not optimal	
Bae Ch	2010	PA soft palate	Histology of local recurrence	Most likely PA recurrence	
Reiland MD	2012	Histology of the upper gingiva PA	Histology of tonsillar PA?	MPA in the skin	
Santaliz-Ruiz LE	2012	No primary hx	No Histology	More suggestive of recurrent PA	
Vivian MA	2012	Histology of primary parotid	Histology of local recurrence	Difficult to appreciate the PA- ONLY Cytology	
Akiba J	2013	Primary palate lesion with vascular invasion	No histology	Met to the maxillary bone- No bone background in the picture	PLAG1 IHC +
RF Chinoy	2013	No primary histology	No histology of recurrence	Suspicious of Epithelial myoepithelial carcinoma	
Tarsitano A	2014	Histology of primary parotid	Histology of local recurrence	The picture shows a lesion underneath the skin, but the image is small	
Abou-Foul AK	2014	Histology of primary parotid	No histology	MPA in the lung	
Mariano FV	2015	Histology of primary parotid	No histology	MPA- good subcutaneous image	
Young VS	2015	Histology of adenocarcinoma of the colon	No histology	PA not showing the background organ	
McGarry JG	2015	Histology of primary parotid	Histology of local recurrence	Suggestive of recurrence	
M Moonim	2015	Histology of primary parotid	Histology of local recurrence	MPA in thyroid	
Knight J	2016	No histology included	No histology	MPA does not show the background organ	
Nakai A	2017	Primary with a capsular irregularity	No recurrence Histology	Histology of MPA in the lung	
Koyama M	2018	Histology of primary parotid	Histology of recurrent multiple	MPA not showing the background organ	
Wasserman JK	2019	Histology of primary parotid	Histology of local recurrence	MPA histology	4 cases total- one with HMGA2-TMCT2 mutation
Wong DKC	2019	Histology of primary parotid	No histology	Difficult to see the image	Infratemporal
Shoukair FL	2020	Histology of the upper lip PA	No histology	Most likely local recurrence	
Fonseca D	2022	Histology of primary parotid	Histology of recurrent multiple	Probably recurrence- difficult to check the lymph node picture	
AlNahwe RW	2023	No histology	No histology	No histology	Quality clinical
W Xu	2023	Histology of primary parotid	No histology	Recurrence- no PA histology picture	No histology picture
Niels Rupp	2023	No histology included	No histology	MPA in the kidney	PLAG1 IHC +
Tsai WH	2024	Histology of primary parotid	Histology of recurrence	Most suggestive of recurrence	

## Conclusion

This systematic review synthesizes current evidence on MPA, demonstrating that, despite its benign histologic appearance, Uncontrolled recurrence is a risk factor for metastatic potential to both regional and distant sites, most notably to bone. These findings underscore the importance of long-term clinical surveillance and meticulous documentation. Furthermore, the review identifies a significant gap in consistent molecular and immunohistochemical profiling. In this regard, incorporating analyses of chromosomal deletions (particularly 9p), as well as markers such as CD105 and *HMGA2::TMTC2*, may enhance understanding of tumor biology and aid in risk stratification. By highlighting these, this study lays the groundwork for future multicenter investigations that integrate molecular diagnostics to improve the accuracy of diagnosis, optimize management strategies, and refine follow-up protocols.

## Data Availability

No datasets were generated or analysed during the current study.

## Code Availability

Statistical Package for the Social Sciences (SPSS) software, version 20.0, ©Copyright IBM (SPSS Inc., Chicago, IL, USA).
